# Genome-Wide Association Study of Plasma Polyunsaturated Fatty Acids in the InCHIANTI Study

**DOI:** 10.1371/journal.pgen.1000338

**Published:** 2009-01-16

**Authors:** Toshiko Tanaka, Jian Shen, Gonçalo R. Abecasis, Aliaksei Kisialiou, Jose M. Ordovas, Jack M. Guralnik, Andrew Singleton, Stefania Bandinelli, Antonio Cherubini, Donna Arnett, Michael Y. Tsai, Luigi Ferrucci

**Affiliations:** 1 Medstar Research Institute, Baltimore, Maryland, United States of America; 2 Clinical Research Branch, National Institute on Aging, Baltimore, Maryland, United States of America; 3 Laboratory of Nutritional Genomics, JM-USDA Human Nutrition Research Center on Aging, Tufts University, Boston, Massachusetts, United States of America; 4 Center for Statistical Genetics, Department of Biostatistics, University of Michigan, Ann Arbor, Michigan, United States of America; 5 Geriatric Rehabilitation Unit, Azienda Sanitaria Firenze (ASF), Florence, Italy; 6 Laboratory of Epidemiology, Demography, and Biometry, National Institute on Aging, Bethesda, Maryland, United States of America; 7 Laboratory of Neurogenetics, National Institute on Aging, Bethesda, Maryland, United States of America; 8 Institute of Gerontology and Geriatrics, Department of Clinical and Experimental Medicine, University of Perugia, Perugia, Italy; 9 Department of Epidemiology, School of Public Health and Clinical Nutrition Research Center, University of Alabama at Birmingham, Birmingham, Alabama, United States of America; 10 Laboratory of Medicine and Pathology, University of Minnesota, Minneapolis, Minnesota, United States of America; University of Liège, Belgium

## Abstract

Polyunsaturated fatty acids (PUFA) have a role in many physiological processes, including energy production, modulation of inflammation, and maintenance of cell membrane integrity. High plasma PUFA concentrations have been shown to have beneficial effects on cardiovascular disease and mortality. To identify genetic contributors of plasma PUFA concentrations, we conducted a genome-wide association study of plasma levels of six omega-3 and omega-6 fatty acids in 1,075 participants in the InCHIANTI study on aging. The strongest evidence for association was observed in a region of chromosome 11 that encodes three fatty acid desaturases (*FADS1*, *FADS2*, *FADS3*). The SNP with the most significant association was rs174537 near *FADS1* in the analysis of arachidonic acid (AA; *p* = 5.95×10^−46^). Minor allele homozygotes had lower AA compared to the major allele homozygotes and rs174537 accounted for 18.6% of the additive variance in AA concentrations. This SNP was also associated with levels of eicosadienoic acid (EDA; *p* = 6.78×10^−9^) and eicosapentanoic acid (EPA; *p* = 1.07×10^−14^). Participants carrying the allele associated with higher AA, EDA, and EPA also had higher low-density lipoprotein (LDL-C) and total cholesterol levels. Outside the *FADS* gene cluster, the strongest region of association mapped to chromosome 6 in the region encoding an elongase of very long fatty acids 2 (*ELOVL2*). In this region, association was observed with EPA (rs953413; *p* = 1.1×10^−6^). The effects of rs174537 were confirmed in an independent sample of 1,076 subjects participating in the GOLDN study. The *ELOVL2* SNP was associated with docosapentanoic and DHA but not with EPA in GOLDN. These findings show that polymorphisms of genes encoding enzymes in the metabolism of PUFA contribute to plasma concentrations of fatty acids.

## Author Summary

Polyunsaturated fatty acids (PUFA) have a number of beneficial effects on human health. Plasma PUFA concentrations are determined by a combination of dietary intake and metabolic efficiency. To determine the genes involved in PUFA homeostasis, we scanned the genome for genetic variations associated with plasma PUFA concentrations. The fatty acid desaturase gene, studied in previous candidate gene association studies, was the strongest determinant of plasma PUFA. A second gene encoding a fatty acid elongase was associated with long chain PUFA. The results of this study contribute to our understanding of the genetics of PUFA homeostasis. These genetic markers may be useful tools to examine the inter-relationship between diet, genetics, and disease.

## Introduction

**Figure 1:**
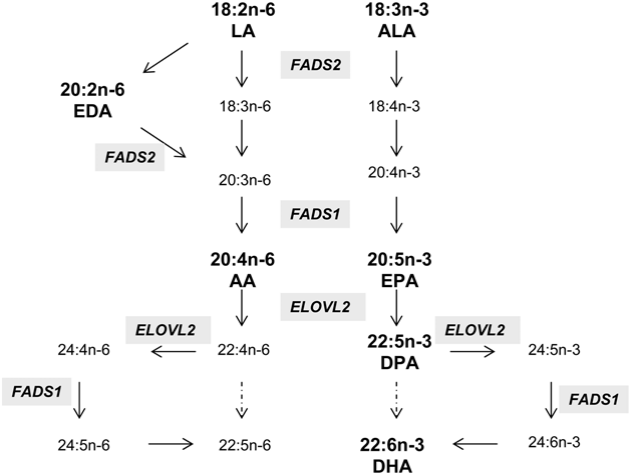
The metabolic pathway of n-3 and n-6 fatty acids. The fatty acids examined in the study are indicated in bold. The dashed arrows indicate pathways absent in mammals.

Polyunsaturated fatty acids (PUFA) refer to the class of fatty acids with multiple desaturations in the aliphatic tail. Short chain PUFA (up to 16 carbons) are synthesized endogenously by fatty acid synthase. Long chain PUFA are fatty acids of 18 carbons or more in length with two or more double bonds. Depending on the position of the first double bond proximate to the methyl end, PUFA are classified as n-6 or n-3. Long chain PUFA are either directly absorbed from food or synthesized from the two essential fatty acids linoleic acid (LA; 18:2n-6) and alpha-linolenic acid (ALA; 18:3n-3) through a series of desaturation and elongation processes [1]. The initial step in PUFA biosynthesis is the desaturation of ALA and LA by the enzyme d6-desaturase (*FADS2*; GeneID 9415) (Figure 1). PUFA modulate inflammatory response through a number of different mechanisms including modulation of cyclooxygenase and lipoxigenase activity [2]. Cyclooxygenase and lipoxigenase are essential for production of eicosanoids and resolvins [2]–[4]. Since n-3 and n-6 fatty acids compete for the same metabolic pathway and produce eicosanoids with differing effects, it has been theorized that the balance of the two classes of PUFA may be important in the pathogenesis of inflammatory diseases.

Epidemiological studies have shown that fatty acid consumption and plasma levels, in particular of the n-3 family, are associated with reduced risk of cardiovascular disease [5]–[7], diabetes [8]–[10], depression [11],[12], and dementia [13]. However, not all studies show significant associations and there has been inconsistencies in the direction of the associations especially for the n-6 acids [14],[15]. The different methods (dietary questionnaire or biomarkers) for accessing PUFA status may contribute to discrepant results [16]–[18]. The disadvantage of using dietary PUFA intake is the evidence of inaccuracies intrinsic in any reporting methods of dietary intake that plasma levels would circumvent. In addition, direct measures of PUFA reflect the cumulative effects of intake and endogenous metabolism. Dietary fatty acids can be converted into longer chain PUFA or stored for energy thus another reason for inconsistent results may be due the general lack of control for individual differences in metabolism once fatty acids are consumed.

Previous studies have examined the association of genetic variants, especially polymorphisms in the *FADS* genes, on fatty acid concentrations in plasma and erythrocyte membranes [19]–[21]. There are 3 *FADS* (*FADS1* [GeneID 3992] ,*FADS2*, and *FADS3* [GeneID 3992]) clustered on chromosome 11. Variants in *FADS1* and *FADS2* have been consistently shown to be associated with PUFA concentrations. It is unknown whether other loci also determine fatty acid concentrations. To address this question, we conducted a genome-wide association study of plasma fatty acid concentration in participants in the InCHIANTI study.

## Results

**Figure 2:**
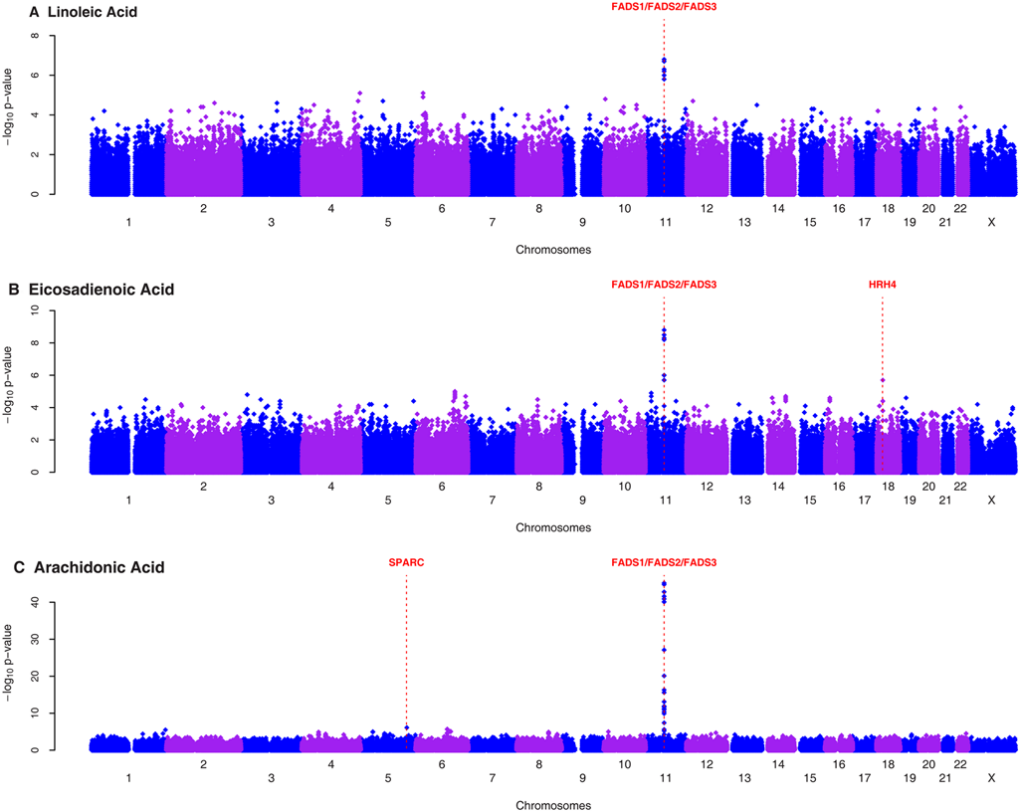
Genome-wide scans of omega-6 fatty acid profiles in InCHIANTI study. Genome-wide associations of plasma linoleic acid (A), eicasadienoic acids (B) and arachidonic acid (C) with 495,343 autosomal and X chromosome SNPs that passed quality control graphed by chromosome position and −log10 p-value. The most significant variant was within the FAD1/FAD2/FAD3 cluster on chromosome 11. The genes nearby or within the SNPs that were selected for replication in GOLDN are indicated.

**Figure 3:**
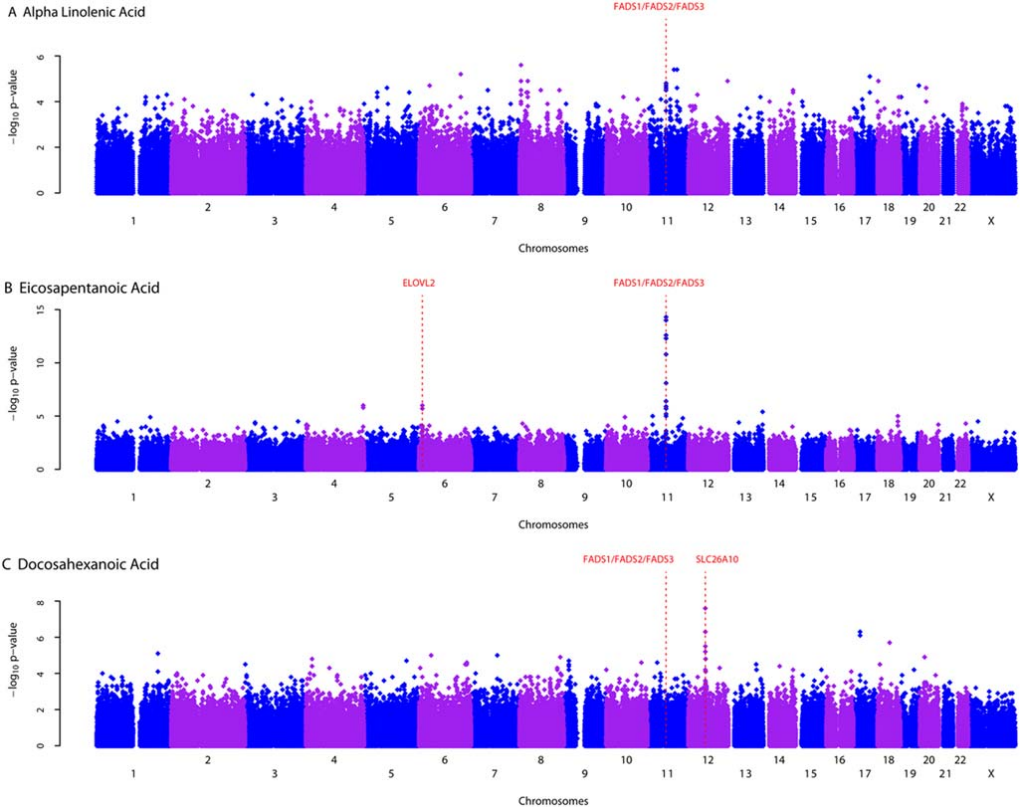
Genome-wide scans of omega-3 fatty acid profiles in InCHIANTI study. Genome-wide associations of plasma alpha linolenic acid (A), eicosapentanoic acid (B) and docasahexanoic acid (C) with 495,343 autosomal and X chromosome SNPs that passed quality control graphed by chromosome position and −log10 p-value. The most significant variant was within the FAD1/FAD2/FAD3 cluster on chromosome 11. The genes nearby or within the SNPs that were selected for replication in GOLDN are indicated.

Linoleic acid (LA) constituted the highest proportion of total fatty acids followed by arachidonic acid (AA) (Table 1) The narrow heritability was highest for AA (37.7%) followed by LA (35.9%), eicosadienoic acid (EDA, 33.3%), alpha-linolenic acid (ALA, 28.1%), eicosapentanoic acid (EPA, 24.4%), and docosahexanoic acid (DHA,12.0%). For EDA, AA, and EPA, genome-wide significant signals fell in the *FADS1/FADS2/FADS3* region on chromosome 11 (Figure 2, Figure 3, Table S1). Of these, the most significant SNP was rs174537 for AA (P = 5.95×10^−46^), where the variant explained 18.6% of the additive variance of AA concentrations. This SNP was significantly associated with EDA (P = 6.78×10^−9^), and EPA (P = 1.04×10^−14^). The association with LA (P = 5.58×10^−7^) and ALA (P = 2.76×10^−5^) did not reach genome-wide significance, and there was no association with DHA (P = 0.3188). Presence of the minor allele (T) was associated with lower concentrations of longer chain fatty acids (EDA, AA, EPA), but with higher concentrations of LA and ALA (Table 2). With the exception of DHA, the SNPs exhibiting the strongest evidence of association with the fatty acids examined in this study mapped to the *FADS1, FADS2,* and *FADS3* cluster. The most significant SNP for DHA was on chromosome 12 within the *SLC26A10* gene (GeneID 65012, rs2277324; P_DHA_ = 2.65×10^−9^). In all cases, inclusion of the most significant SNP as a covariate in the model resulted in attenuation of the effect of the other SNPs in the region (Figure S1). Accordingly, associated SNPs in this region were in significant linkage disequilibrium with each other in the InCHIANTI sample (Figure S2).

**Table 1:** Descriptive Characteristics of InCHIANTI and GOLDN study.

Trait	INCHIANTI		GOLDN	
N (m/f)	1075	(485/590)	1076	(519/557)
Age (years)	68.37	(15.5)	48.4	(16.4)
BMI (kg/m2)	27.12	(4.1)	28.3	(5.6)
Total Cholesterol (mg/dL)	213.62	(40.7)	190.1	(38.9)
HDL Cholesterol (mg/dL)	55.98	(15.1)	47	(13.1)
LDL Cholesterol (mg/dL)	133.08	(35.3)	121	(31.3)
Triglyceride (mg/dL)	122.79	(65.1)	139.2	(117.3)
Glucose (mg/dl)	94.23	(26.2)	101.6	(19.0)
Linoleic Acid^a^	24.8	(4.0)	12.9	(1.4)
Linolenic Acid^a^	0.4	(0.3)	0.1	(0.0)
Eicosadienoic Acid^a^	0.1	(0.1)	N/A	
Arachidonic Acid^a^	8.0	(1.9)	13.6	(1.2)
Eicosapentanoic Acid^a^	0.61	(0.2)	0.5	(0.3)
Docosahexanoic Acid^a^	2.29	(0.8)	3.0	(0.9)
Total energy , kal/day	2000	(596)	2122	(1190)
Dietary fat, % energy	30.9	(5.1)	35.4	(6.9)

Values represent mean (SD).

a Fatty acids are plasma concentrations (% total fatty acids) for InCHIANTI and erythrocytes concentration for GOLDN.

**Table 2:** Associations of fatty acids and plasma lipids by rs174537 (*FADS1*) and rs953413 (*ELOVL2*) in InCHIANTI and GOLDN study.

	InCHIANTI							GOLDN						
FADS: rs174537	G/G (n = 569)		T/G (n = 414)		T/T (n = 92)		P	G/G (n = 433)		T/G (n = 495)		T/T (n = 139)		P
Linoleic acid	24.27	(3.99)	25.24	(3.98)	25.88	(3.69)	<0.0001	11.97	(0.15)	12.46	(0.14)	13.49	(0.17)	<0.0001
Eicosadienoic acid	0.12	(0.05)	0.11	(0.06)	0.09	(0.07)	<0.0001							
Arachidonic acid	8.72	(1.86)	7.39	(1.51)	6.35	(1.36)	<0.0001	14.06	(0.09)	13.45	(0.09)	12.43	(0.12)	<0.0001
Alpha Linolenic acid	0.42	(0.24)	0.47	(0.26)	0.46	(0.22)	<0.0001	0.14	(0)	0.14	(0)	0.16	(0)	<0.0001
Eicosapentanoic acid	0.65	(0.24)	0.58	(0.18)	0.51	(0.16)	<0.0001	0.49	(0.02)	0.47	(0.02)	0.42	(0.03)	<0.0001
Docosapentanoic acid								1.97	(0.03)	1.93	(0.03)	1.83	(0.03)	<0.0001
Docosahexanoic acid	2.31	(0.77)	2.29	(0.76)	2.23	(0.77)	0.3188	3.28	(0.08)	3.22	(0.08)	3.13	(0.1)	0.0677
Triglyceride (mg/dL)	127.5	(84.2)	120.5	(61)	139.3	(100.4)	0.862	211	(18.7)	211.1	(19.8)	228.5	(31.6)	0.369
Total Cholesterol (mg/dL)	217	(40.9)	212.5	(40.2)	209.3	(40.4)	0.0268	202.5	(3.7)	194.2	(3.3)	194.4	(4.1)	0.0007
HDL Cholesterol (mg/dL)	55.8	(15.5)	56.2	(14.4)	54.6	(13.9)	0.7752	46.2	(1.3)	45.7	(1.3)	45.9	(1.4)	0.801
LDL Cholesterol (mg/dL)	135.6	(35.3)	132.2	(35)	126.9	(37.3)	0.0112	124.3	(3.2)	118.3	(2.9)	118	(3.2)	0.0093
**ELOVL2: rs953413**	**G/G (n = 343)**		**A/G (n = 520)**		**A/A (n = 211)**			**G/G (n = 344)**		**A/G (n = 527)**		**A/A (n = 190)**		
Eicosapentanoic acid	0.58	(0.25)	0.61	(0.18)	0.66	(0.22)	<0.0001	0.48	(0.02)	0.47	(0.03)	0.48	(0.03)	0.1817
Docosapentanoic acid								1.89	(0.03)	1.94	(0.03)	1.99	(0.03)	0.0007
Docosahexanoic acid	2.37	(0.79)	2.29	(0.75)	2.17	(0.74)	0.0044	3.32	(0.08)	3.2	(0.08)	3.1	(0.1)	0.0018

Values represent mean (SD).

Fatty acids are plasma concentrations (% total fatty acids) for InCHIANTI and erythrocytes concentration for GOLDN.

To investigate whether this SNP has an effect on other cardiovascular disease risk factors, we examined the association of rs174537 with plasma lipid parameters. Significant association was observed with total cholesterol (P = 0.027) and LDL-cholesterol (P = 0.011), but not with either HDL-C (P = 0.775) or triglycerides (P = 0.862; Table 2). The minor allele homozygotes (TT) had 8 mg/dL lower total cholesterol and 9 mg/dL lower LDL-C compared with GG subjects.

To identify other putative chromosome regions associated with fatty acid concentrations beyond the *FADS* cluster, we examined the top 3 non-redundant (r2<0.2) SNPs from the analysis of each fatty acid and selected the SNPs that mapped to candidate gene regions (defined as 20kb upstream of intron 1, or downstream of last exon) for replication in an independent study (Table S1). These included rs16940765 (*HRH4* [GeneID 59340], chr18, P_EDA_ = 2.18×10^−6^), rs17718324 (*SPARC* [GeneID 6678], chr5, P_AA_ = 7.64×10^−7^) and rs953413 (*ELOVL2* [GeneID 54898], chr6, P_AA_ = 1.1×10^−6^). In the InCHIANTI study, these four SNPs most strongly associated with a specific fatty acid, unlike *FADS* cluster that was associated with multiple fatty acids. We noted, however that rs16940765 (*HRH4*), rs953413 (*ELOVL2*) and rs2277324 (*SLC26A10*) showed significant association at the 0.05 level for AA (P = 0.003), DHA (0.004), and EPA (P = 0.004) respectively. In addition, there were four SNPs (rs953413, rs1570069, rs3798719, rs7744440) in the *ELOVL2* gene were associated with EPA with p values ranging from 9.51×10^−5^ to 1.10×10^−6^ (Figure S3). In total, 5 SNPs (rs174537, rs2277324, rs16940765, rs17718324, rs953413) were selected for replication.

In the GOLDN study, there were significant associations of *FADS* SNP, rs174537, with ALA, LA, AA, EPA and DHA (P<0.001) and marginal association with docosapentaenoic acid (DPA) (P = 0.068) (Table 2 **)**. As with the InCHIANTI study, presence of the T allele was associated with higher ALA and LA concentration and lower AA, EPA, DPA and DHA concentrations. Consistent with the InCHIANTI study, this SNP was associated with total cholesterol and LDL-C but not triglycerides or HDL-C. We also observed strong associations of rs953413 with docosapentanoic acid (DPA; P = 0.002) and DHA (P<0.001). The presence of the minor allele (A) was associated with lower DHA and higher DPA and higher AA compared to the minor allele carriers (Table 2). The remaining 3 SNP (rs2277324, rs16940765, rs17718324) were not associated with fatty acid concentrations in the GOLDN study (data not shown).

## Discussion

The genome-wide association approach enables comprehensive examination of the genome to identify novel loci contributing to PUFA homeostasis. In addition, the significance of the genes previously reported in association with PUFA can be assessed relative to other regions in the genome. Here, we demonstrated that polymorphisms in the *FADS* cluster are the strongest determinants of plasma and erythrocyte fatty acid concentrations, explaining up to 18.6% of the additive variance in plasma AA levels. Consistent with prior reports, the greatest evidence of association was observed in the region containing *FADS1*, *FEN1* (flap structure specific endonuclease, GeneID 2237), two hypothetical proteins (*C11orf9* [GeneID 745], *C11orf10* [GeneID 746]), and the promoter region of *FADS2* [20],[21]. With the exception of EDA, the direction of the association of rs174537 with plasma and erythrocyte fatty acids is consistent with previous reports. We find that there are higher levels of ALA and LA which is suggestive of an accumulation of the initial products of the PUFA metabolic pathway. The cluster of SNP ranging from rs174537 to rs509360 showed the strongest evidence for association, and contains the haplotype block previously examined in relation to plasma and erythrocyte fatty acids [20],[21]. Based on the HapMap CEU data, the r2 between rs174537 and previously reported SNPs were ≥0.8. If functional polymorphisms exist within this region, it could affect the expression of both desaturases. To this end, in a recent report of genome-wide association of global gene expression, the rs174546 that is in LD with the rs174537 (r^2^ = 0.99) was associated with FADS1 expression (LOD = 5, P = 1.6×10^−6^) but not FADS2 (LOD = 0.7, P = 0.07) in lymphoblastoid cells [22],[23]. The allele associated with higher AA showed greater expression of FADS1, consistent with our results. Since *FADS1* and *FADS2*expression varies by tissue type, it would be of interest to examine the effect of the variant on gene expression in other cell types [24].

The T allele associated with lower AA was also associated with decreased LDL-C and total cholesterol. The association with LDL-C was also observed in a large meta-analysis of plasma lipid concentrations in ∼8500 subjects [25],[26]. In this meta-analysis, there was stronger evidence of association with high density lipoprotein (HDL-C) and triglycerides (TG), where the T allele displayed lower HDL-C and higher triglyceride concentrations. Finally, in the Welcome Trust Case-Control Consortium coronary artery disease (CAD) study, the T allele was associated with increased prevalence of CAD (P = 0.0375) [27]. The increased prevalence of CAD, low HDL-C and high TG is consistent with lower AA concentrations in the T allele carriers. Endogenous PUFA are natural ligands of peroxisome proliferator activating receptor alpha (PPARA) [28]. PPARA activation has been shown to elevate HDL-C and lower TG by inducing the expression of ApoA1, Apo-AII, lipoprotein lipase and suppressing ApoCIII [29]–[32]. Thus the low AA, EPA and EDA in the T allele carriers will results in lower PPARA activation. Under this hypothesis, we would expect the T allele carriers to display higher LDL-C since PPARA is known to enhance LDL-C clearance [33] . However, in both the InCHIANTI and GOLDN study, lower concentrations of LDL-C are observed. It is likely that there are other mechanisms by which fatty acids regulate lipoprotein homeostasis, for example through membrane fluidity. It may be possible, that the higher concentrations of linoleic and linolenic acid in the T allele carriers results in increased membrane fluidity, thus increasing LDL-receptor recycling leading to lower LDL-C.

The elongation of very long chain fatty acid (*ELOVL*) family genes are elongases that catalyze the rate-limiting condensation reaction resulting in the synthesis of very long chain fatty acids (VLCFA) [34]. To date, six *ELOVL* genes have been described. The ELOVL1, 3 and 6 are involved in synthesis of monounsaturated and saturated long chain fatty acids while ELOVL2, 4 and 5 elongate polyunsaturated fatty acids [35]. In this study, rs953413 in the *ELOVL2* was the third most significant SNP in the analysis of EPA, with strong, although not genome-wide level significant association with long chain fatty acids EPA and DHA. In GOLDN, there were no significant associations of this SNP with EPA, but a significant association was observed with DPA. In mammals, two elongation steps are required for the synthesis of DHA from EPA. First, EPA is elongated to DPA, then to 24:5n-3 followed by a desaturation and retroconversion step to form DHA [1] (Figure 1). The two initial elongation steps of 20 and 22-C fatty acids are mediated by ELOVL2 [36]. The rs953413 is associated with substrate EPA (InCHIANTI), and product DPA (GOLDN) and DHA (both studies) of the EVOLV2 pathway. Plasma DPA levels were not measured in InCHIANTI, thus whether this association is also observed in this population cannot be investigated. Why the *ELOVL2* SNP was not associated with EPA in GOLDN is not clear, however it may reflect the differences in fatty acid metabolism in erythrocytes versus plasma as they reflect two slightly different pools of fatty acids [37]. Plasma fatty acids reflect short term intake of fatty acids whereas erythrocyte levels reflect long term intake. Thus the different results between the plasma and erythrocyte fatty acids may reflect dietary differences between subjects in the GOLDN (USA) and the InCHIANTI study (Italy). Regardless of these differences, the results of this study suggestive of the role of *ELOVL2* in the conversion of EPA to DHA in humans. The presence of the minor (A) allele was associated with higher EPA/DPA and lower DHA. If rs953413, located in intron 1, is the functional SNP (or is in LD with the functional SNP), this variant would likely be associated with lower expression of the *ELOVL2* or result in a less efficient variant of the elongase resulting in decreased elongation of EPA to DHA. In lymphoblastoid cells, this SNP was not associated with *ELOVL2* expression (LOD = 0.4, P = 0.2) [22],[23]. Further investigation in other cell lines and functional analysis of the different variants is warranted.

In summary, we have shown that the major loci for fatty acid concentrations in both plasma and erythrocyte membranes are in genes involved in the metabolism of PUFA. The FADS locus on chromosome 11 was the major contributor of plasma fatty acid concentrations, and thus may have implications for cardiovascular disease. In addition, we have identified a second promising locus in *ELOVL2* that is involved in the homeostasis of longer chain n-3 fatty acids. Future studies should investigate the interactions between dietary intake, circulating levels of fatty acids and genetic variants on risk of diseases such as cardiovascular disease.

## Material and Methods

### Sample Description

The InCHIANTI study is a population-based epidemiological study aimed at evaluating factors that influence mobility in the older population living in the Chianti region of Tuscany, Italy. Details of the study have been previously reported [38]. Briefly, 1616 residents were selected from the population registry of Greve in Chianti (a rural area: 11 709 residents with 19.3% of the population greater than 65 years of age) and Bagno a Ripoli (Antella village near Florence; 4704 inhabitants, with 20.3% greater than 65 years of age). The participation rate was 90% (n = 1453) and participants ranged between 21–102 years of age. Overnight fasted blood samples were collected for genomic DNA extraction and measurement of plasma fatty acids. Genotyping was completed for 1231 subjects using the Illumina Infinium HumanHap 550 genotyping chip (ver1 and ver3 chips were used). The study protocol was approved by the Italian National Institute of Research and Care of Aging Institutional Review.

There were 85 parent-offspring pairs, 6 sib-pairs and 2 half-sibling pairs documented. We investigated any further familial relationships using IBD of 10,000 random SNPs using RELPAIR and uncovered 1 parent-offspring, 79 siblings and 13 half-sibling [39]. We utilized the correct family structure inferred from genetic data for all analyses. In addition, we identified 2 duplicated samples and removed these from the study. Sample quality was assessed using the GAINQC program (http://www.sph.umich.edu/csg/abecasis/GainQC/). The average genotype completeness and heterozygosity rates were 98% and 32% respectively. We excluded subjects that had less than 97% of genotyped completeness (n = 12), heterozygosity rate of less than 30% (n = 5) and misspecified sex based on heterozygosity of the X chromosome SNPs (n = 1). The final sample size used for SNP quality control was 1210.

The confirmation study population consisted of 1120 white men and women in the United States participating in the Genetics of Lipid Lowering Drugs and Diet Network (GOLDN) Study. The majority of participants were re-recruited from the ongoing National Heart and Lung and Blood Institutes (NHLBI) Family Heart Study (FHS) [40] in two genetically homogeneous centers (Minneapolis, MN and Salt Lake City, UT). GOLDN is part of the Program for Genetic Interactions (PROGENI) Network, a group of NIH-funded intervention studies of gene-environmental interactions. The primary aim of the GOLDN study was to characterize the genetic components of triglycerides response following a high fat meal and hypolipedemic drug, fenofibrate. Detailed study design and methodology has been previously described [41],[42].

In the replication sample, we excluded persons with missing genotypes or extreme fatty acid values. The final data set consists of information on 1076 individuals. The protocol for this study was approved by the Human Studies Committee of Institutional Review Board at University of Minnesota, University of Utah and Tufts University/New England Medical Center. Written informed consent was obtained from all participants.

### Biochemical Measurements

*InCHIANTI:* Plasma fatty acid measurement methods has been described previously [43]. Briefly, blood samples were collected in the morning after a 12-hr overnight fast. Aliquots of plasma were immediately obtained and stored at −80 C. Fatty acid methyl esters (FAME) were prepared through transesterification using Lepage and Roy’s method with modification [44],[45]. Separation of FAME was carried out on an HP-6890 gas chromatograph (Hewlett- Packard, Palo Alto, CA) with a 30-m fused silica column (HP-225; Hewlett-Packard). FAMEs were identified by comparison with pure standards (NU Chek Prep, Inc., Elysian, MA). For quantitative analysis of fatty acids as methyl esters, calibration curves for FAME (ranging from C14:0 to C24:1) were prepared by adding six increasing amounts of individual FAME standards to the same amount of internal standard (C17:0; 50xg). The correlation coefficients for the calibration curves of fatty acids were in all cases higher than 0.998 in the range of concentrations studied. Fatty acid concentration was expressed as a percentage of total fatty acids. The coefficient of variation for all fatty acids was on average 1.6% for intraassay and 3.3% for interassay. HDL-C, total cholesterol and triglycerides were determined using commercial enzymatic tests (Roche Diagnostics, Mannheim, Germany). Serum low-density lipoprotein cholesterol (LDL-C) was computed with the Friedewald formula (LDL-C = total cholesterol − HDL-C − triglicerides/5).

*GOLDN*: Fatty acids (FA) in erythrocyte membrane were measured following procedures described previously [46] Briefly, lipids were extracted from the erythrocyte membranes with a mixture of chloroform:methanol (2:1, v/v), collected in heptanes and injected onto a capillary Varian CP7420 100-m column with a Hewlett Packard 5890 gas chromatograph (GC) equipped with a HP6890A autosampler. The GC was configured for a single capillary column with a flame ionization detector and interfaced with HP chemstation software. The initial temperature of 190°C was increased to 240°C over 50 minutes. Fatty acid methylesters from 12:0 through 24:1n9 were separated, identified and expressed as percent of total fatty acid. Triglycerides were measured using a glycerol blanked enzymatic method (Trig/GB, Roche Diagnostics Corporation, Indianapolis, IN) and cholesterol was measured using a cholesterol esterase, cholesterol oxidase reaction (Chol R1, Roche Diagnostics Corporation) on the Roche/Hitachi 911 Automatic Analyzer (Roche Diagnostics Corporation). For HDL-cholesterol, the non-HDL-cholesterol was first precipitated with magnesium/dextran. LDL-cholesterol was measured by a homogeneous direct method (LDL Direct Liquid Select Cholesterol Reagent, Equal Diagnostics, Exton, PA).

### Assessment of Dietary Intake

In the InCHIANTI, dietary intake was assessed using a food-frequency questionnaire (FFQ) created for the European Prospective Investigation into Cancer and nutrition (EPIC) study, and has previously been validated to provide good estimates of dietary intake in this study population [47],[48]. In GOLDN, habitual dietary intake was estimated using the validated dietary history questionnaire (DHQ) developed by the National Cancer Institute [49]. We excluded subjects that reported <800 kcal and >5500 kcal in men and <600kcal and >4500kcal in women.

### Genotyping

*InCHIANTI*: Genome-wide genotyping was performed using the Illumina Infinium HumanHap550 genotyping chip (chip version 1 and 3) as previously described [50]. The SNP quality control was assessed using GAINQC. The exclusion criteria for SNPs were minor allele frequency <1% (n = 25,422), genotyping completeness <99% (n = 23,610) and Hardy Weinberg-equilibrium (HWE) <0.0001 (n = 517). 

*GOLDN*: Five SNPs were selected for replication in the GOLDN study: rs953413, rs2277324, rs16940765, rs17718324 and rs174537. One of these, rs2277324, failed genotyping and therefore another SNP in high LD, rs923838 (r^2^ = 0.89 in hapmap), was used as a proxy for this SNP. DNA was extracted from blood samples and purified using commercial Puregene reagents (Gentra System, Inc.) following manufacturer’s instructions. SNPs were genotyped using the 5’nuclease allelic discrimination Taqman assay with allelic specific probes on the ABI Prism 7900HT Sequence Detection System (Applies Biosystems, Foster City, Calif, USA) according to standard laboratory protocols. The primers and probes were pre-designed (the assay -on -demand) by the manufacturer (Applied Biosystem) (Assay ID: FEN_rs174537: C___2269026_10, HRH4_rs16940765: C__32711739_10, SPARC_rs17718324: C__34334455_10, ELOVL2_rs953413: C___7617198_10, rs923828: C___2022671_10).

### Statistical Analysis

*InCHIANTI GWAS*: Inverse normal transformation was applied to plasma fatty acid concentrations to avoid inflated type I error due to non-normality [51]. The genotypes were coded 0, 1 and 2 reflecting the number of copies of an allele being tested (additive genetic model). For X-chromosome analysis, the average phenotype of males hemizygous for a particular allele was treated assumed to match the average phenotype of females homozygous for the same allele. Association analysis was conducted by fitting simple regression test using the fastAssoc option in MERLIN [52]. Narrow heritability reflects the ratio of the trait’s additive variance to the total variance [51],[53]. In all the analyses, the models were adjusted for sex, age and age squared. The genomic control method was used to control for effects of population structure and cryptic relatedness [54]. An approximate genome-wide significance threshold of 1×10^−7^ (∼0.05/495343 SNPs) was used. For each fatty acid concentration, a second analysis included the most significant SNP from the first pass analysis as a covariate. Linkage disequilibrium coefficints within the region of interest were calculated using GOLD [55].

For the other phenotypes (total cholesterol, triglycerides, LDL-cholesterol, HDL-cholesterol and BMI), the traits were normalized either by natural log or square root transformation when necessary. Associations for each SNP were investigated using the general linear model (GLM) procedure in SAS.

*GOLDN:* Inverse normal transformation was applied to erythrocyte membrane fatty acid concentration to achieve approximate normality. For the additive model, genotype coding was based on the number of variant alleles at the polymorphic site. With no significant sex modification observed, men and women were analyzed together. We used the generalized estimating equation (GEE) linear regression with exchangeable correlation structure as implemented in the GENMOD procedure in SAS (Windows version 9.0, SAS Institute, Cary, NC) to adjust for correlated observations due to familial relationships. Potential confounding factors included study center, age, sex, BMI, smoking (never, former and current smoker), alcohol consumption (non-drinker and current drinker), physical activity, drugs for lowering cholesterol, diabetes and hypertension and hormones. A two-tailed *P* value of <0.05 was considered to be statistically significant.

## Supporting Information

Figure S1 Q-Q plots for (A) linolenic acid, (B) eicosadienoic acid (C) arachidonic acid, (D), alpha-linolenic acid, (E), eicsapentanoic acid, and (F) docsahexanoic acid from the first analysis (red circles) and the second analysis after including the most significant SNP (blue circles).
(0.52 MB TIF)

Figure S2 The associations in the fatty acid desaturase clusters on chromosome 11 are displayed. (A) The −log10 pvalues for each fatty acid concentration within the FADS cluster on chromosome 11. The y axis is truncated at 14, the most significant SNP for arachidonic acid rs174537 at −log10 value of 45. (B) The genes that lie +/− 100kb of rs174537 and (C) pairwise LD (r^2^) in the region ranging from high (red), intermediate (green), to low (blue) in the InCHIANTI study.
(0.76 MB TIF)

Figure S3 The associations in the elongation of very long fatty acid 2 gene are displayed. (A) The −log10 pvalues for each fatty acid concentration around the ELOVL2 gene. (B) The genes that lie +/− 100kb of rs953413 and (C) pairwise LD (r^2^) in the region ranging from high (red), intermediate (green), to low (blue) in the InCHIANTI study.
(0.53 MB TIF)

Table S1 Top 10 non-redundant SNPs for each plasma fatty acid concentrations.
(0.15 MB DOC)
