# Supplementary figures and images for: Genome-Wide Association Study of Plasma Polyunsaturated Fatty Acids in the InCHIANTI Study
Source: PLoS Genet. 2009 Jan 16;5(1):e1000338. doi: 10.1371/journal.pgen.1000338 (PMC2613033; doi:10.1371/journal.pgen.1000338)

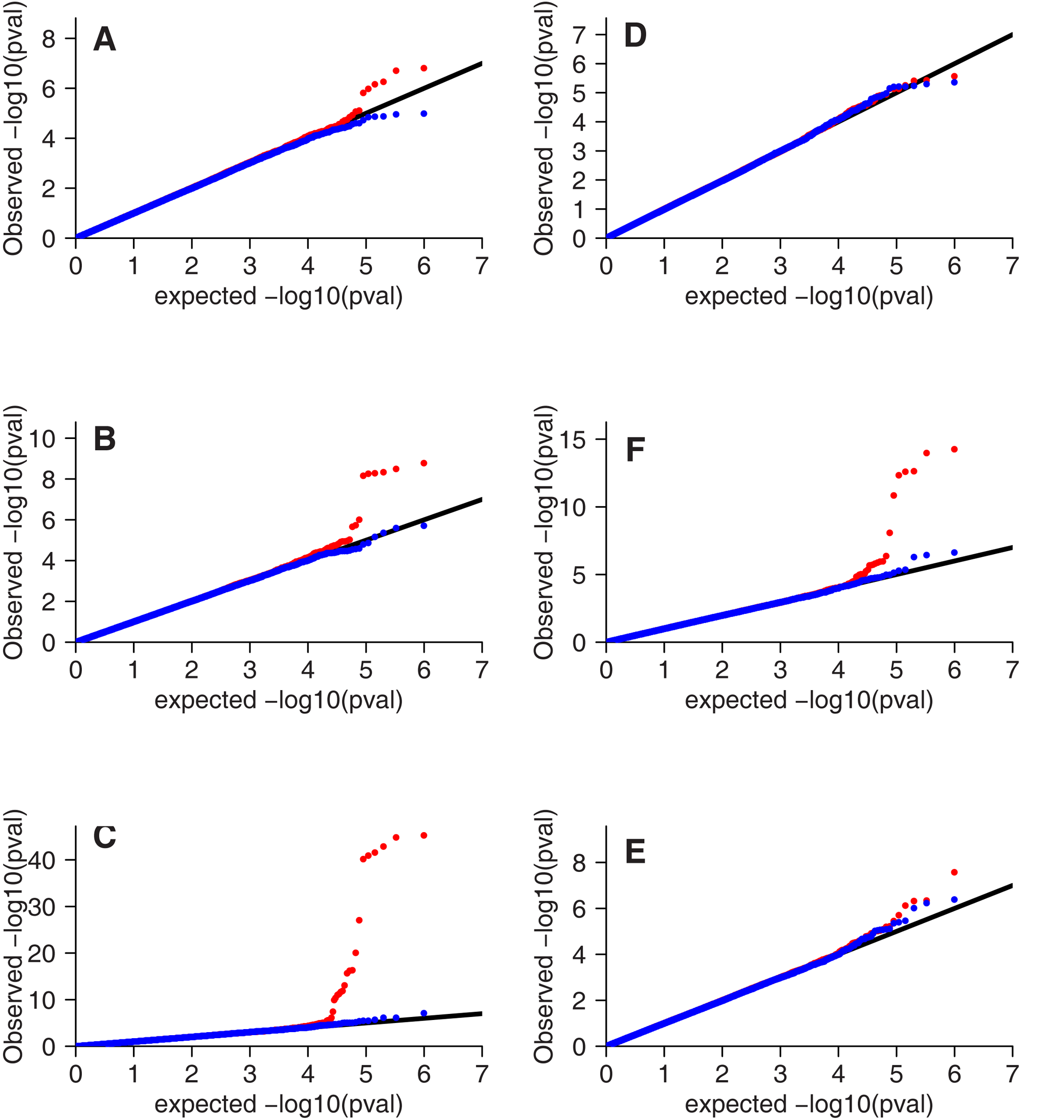

Supplement: Figure S1 — Q-Q plots for (A) linolenic acid, (B) eicosadienoic acid (C) arachidonic acid, (D), alpha-linolenic acid, (E), eicsapentanoic acid, and (F) docsahexanoic acid from the first analysis (red circles) and the second analysis after including the most significant SNP (blue circles). (0.52 MB TIF) [file pgen.1000338.s001.tif]

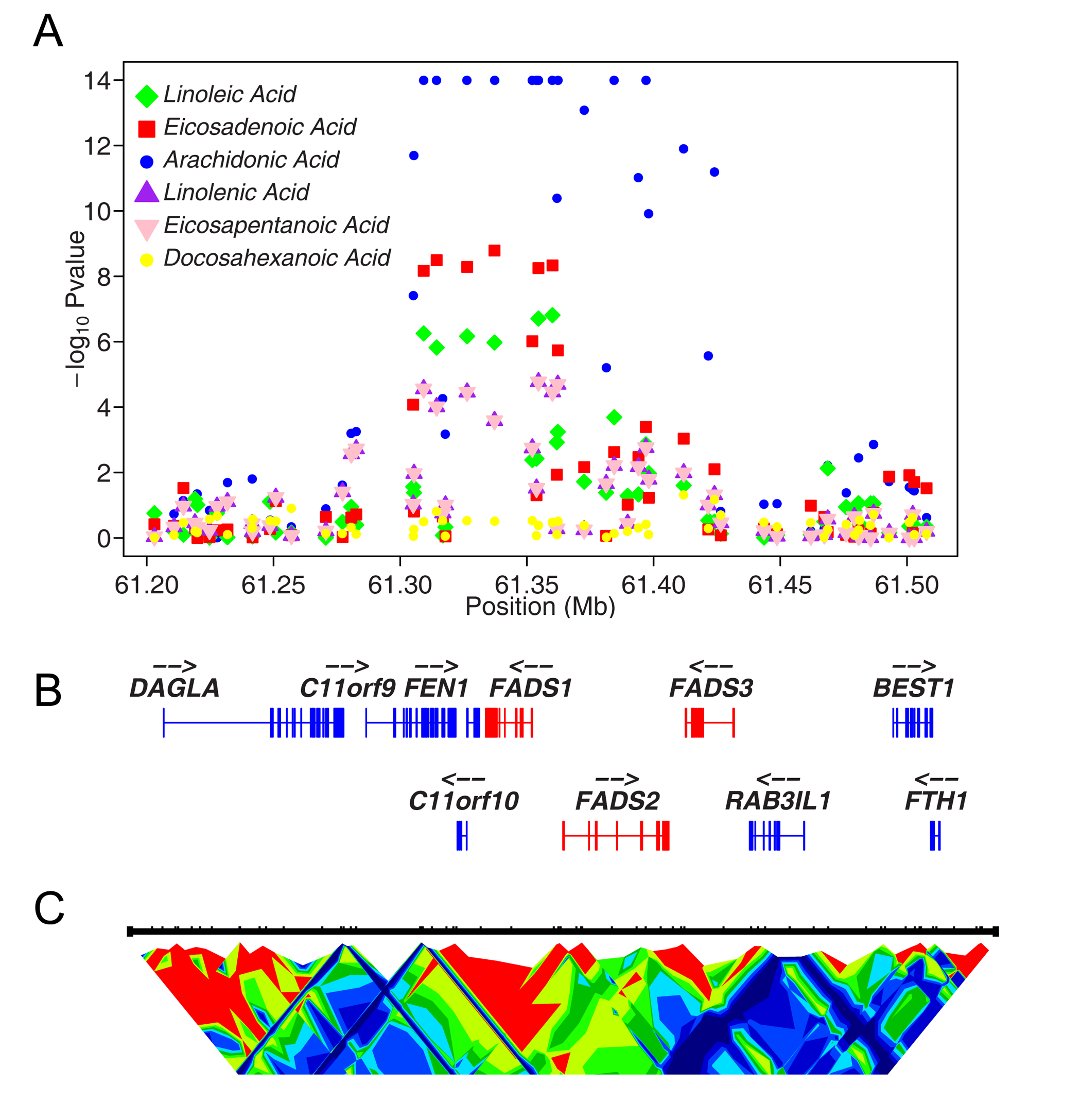

Supplement: Figure S2 — The associations in the fatty acid desaturase clusters on chromosome 11 are displayed. (A) The −log10 pvalues for each fatty acid concentration within the FADS cluster on chromosome 11. The y axis is truncated at 14, the most significant SNP for arachidonic acid rs174537 at −log10 value of 45. (B) The genes that lie +/− 100kb of rs174537 and (C) pairwise LD (r2) in the region ranging from high (red), intermediate (green), to low (blue) in the InCHIANTI study. (0.76 MB TIF) [file pgen.1000338.s002.tif]

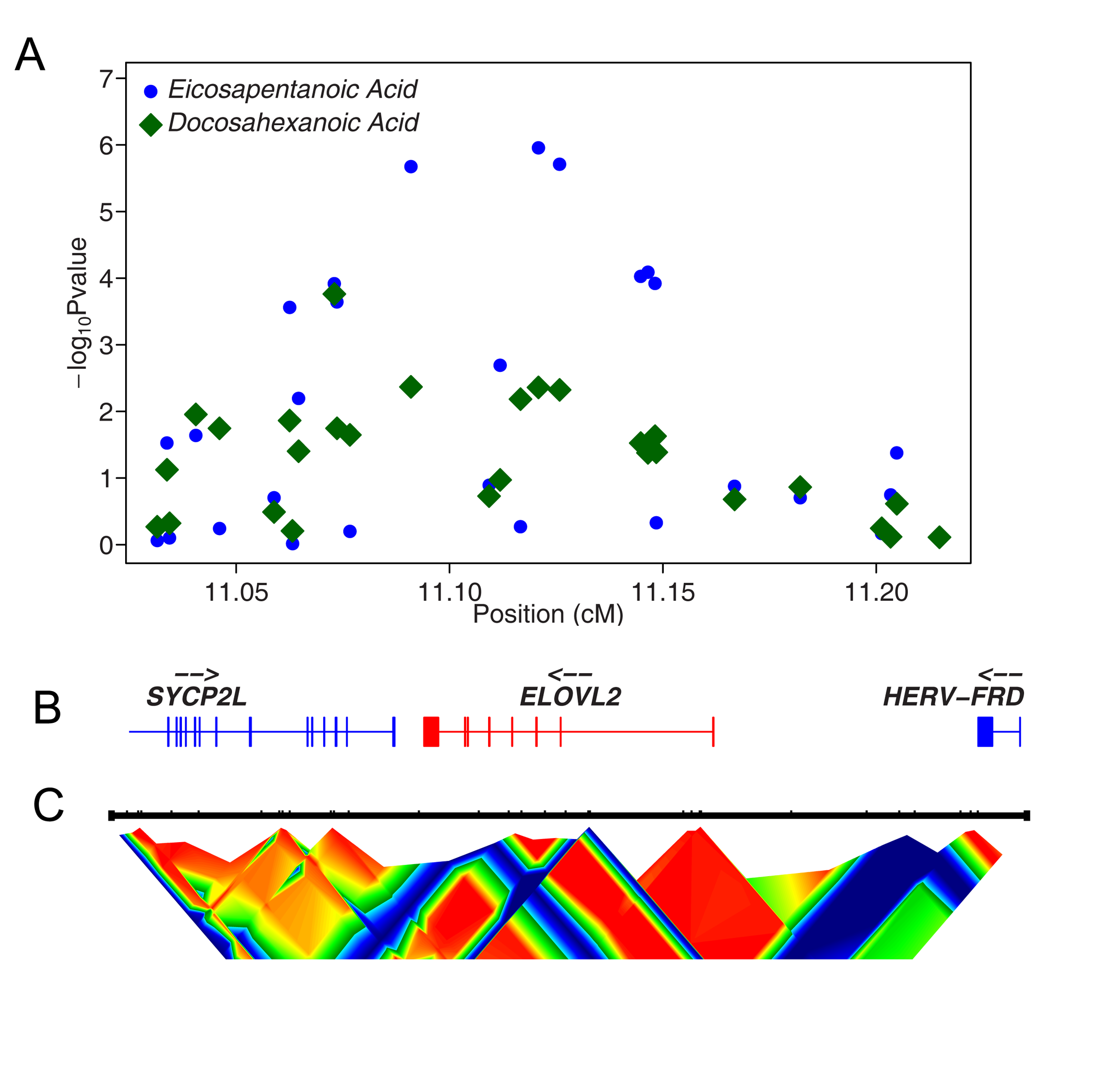

Supplement: Figure S3 — The associations in the elongation of very long fatty acid 2 gene are displayed. (A) The −log10 pvalues for each fatty acid concentration around the ELOVL2 gene. (B) The genes that lie +/− 100kb of rs953413 and (C) pairwise LD (r2) in the region ranging from high (red), intermediate (green), to low (blue) in the InCHIANTI study. (0.53 MB TIF) [file pgen.1000338.s003.tif]
