# Supplementary material for: Genome-Wide Association Study of Plasma Polyunsaturated Fatty Acids in the InCHIANTI Study
Source: PLoS Genet. 2009 Jan 16;5(1):e1000338. doi: 10.1371/journal.pgen.1000338 (PMC2613033; doi:10.1371/journal.pgen.1000338)
Supplement: Table S1 — Top 10 non-redundant SNPs for each plasma fatty acid concentrations. (0.15 MB DOC) [file pgen.1000338.s004.doc]

**Table S1**. **Top 10 non-redundant SNPs for each plasma fatty acid concentrations.**

| **Trait** | **CHR** | **Position (bp)** | **SNP** | **Alleles  (+/-)** | **Freq (+)** | **EFFECT**^1^ | **H2**^2^ | **P-value** |
| --- | --- | --- | --- | --- | --- | --- | --- | --- |
| Linoleic Acid | 11 | 61360086 | rs174576 | C/a | 0.71 | -0.22 | 2.46 | 1.54E-07 |
|  | 4 | 189339095 | rs13435183 | C/t | 0.87 | 0.26 | 1.90 | 7.73E-06 |
|  | 6 | 23482193 | rs6918629 | A/g | 0.60 | 0.18 | 1.94 | 8.50E-06 |
|  | 10 | 4223037 | rs7922038 | C/t | 0.66 | 0.18 | 1.81 | 1.42E-05 |
|  | 5 | 73748965 | rs435958 | C/t | 0.56 | -0.17 | 1.67 | 1.81E-05 |
|  | 12 | 20488753 | rs10841528 | A/g | 0.59 | 0.17 | 1.69 | 1.93E-05 |
|  | 4 | 183293713 | rs12503812 | G/a | 0.80 | 0.21 | 1.67 | 2.19E-05 |
|  | 3 | 117997046 | rs16825624 | T/c | 0.94 | 0.35 | 1.61 | 2.71E-05 |
|  | 2 | 156750389 | rs10497175 | G/t | 0.76 | 0.19 | 1.68 | 2.80E-05 |
|  | 4 | 39887129 | rs7674515 | C/t | 0.94 | 0.34 | 1.52 | 3.22E-05 |
| Eicosadienoic Acid | 11 | 61337211 | rs174556 | C/t | 0.76 | 0.30 | 3.30 | 1.63E-09 |
|  | 18 | 20302545 | rs16940765^┼^ | T/c | 0.93 | -0.39 | 2.08 | 2.18E-06 |
|  | 6 | 127518410 | rs2489629 | C/t | 0.53 | 0.20 | 1.95 | 9.34E-06 |
|  | 11 | 19843265 | rs11025300 | G/a | 0.84 | -0.26 | 1.80 | 1.15E-05 |
|  | 3 | 21276174 | rs9815041 | C/a | 0.62 | 0.20 | 1.79 | 1.60E-05 |
|  | 6 | 162874576 | rs4493732 | C/t | 0.54 | 0.19 | 1.73 | 1.81E-05 |
|  | 14 | 77565785 | rs11622927 | C/a | 0.89 | 0.29 | 1.69 | 1.87E-05 |
|  | 16 | 15036960 | rs4985155 | T/c | 0.67 | -0.19 | 1.68 | 2.35E-05 |
|  | 19 | 20613342 | rs8100262 | T/g | 0.67 | 0.20 | 1.75 | 2.37E-05 |
|  | 14 | 33279026 | rs12880943 | T/c | 0.87 | -0.27 | 1.62 | 2.78E-05 |
| Arachidonic Acid | 11 | 61309256 | rs174537^┼^ | G/t | 0.72 | 0.67 | 18.56 | 5.95E-46 |
|  | 5 | 151038372 | rs17718324^┼^ | G/a | 0.93 | -0.43 | 2.32 | 7.64E-07 |
|  | 6 | 102272022 | rs1222955 | A/g | 0.82 | 0.26 | 2.10 | 1.91E-06 |
|  | 11 | 61421568 | rs13966 | T/c | 0.52 | 0.21 | 2.20 | 2.70E-06 |
|  | 1 | 241947159 | rs12035552 | C/t | 0.64 | 0.21 | 2.01 | 3.42E-06 |
|  | 6 | 113742923 | rs2842846 | A/c | 0.75 | -0.22 | 1.82 | 8.13E-06 |
|  | 10 | 14571536 | rs10508474 | C/t | 0.87 | 0.29 | 1.90 | 1.02E-05 |
|  | 5 | 40129389 | rs1857869 | A/g | 0.88 | 0.28 | 1.83 | 1.08E-05 |
|  | 4 | 54352841 | rs4864809 | G/a | 0.62 | 0.20 | 1.88 | 1.13E-05 |
|  | 8 | 104247557 | rs10099640 | C/t | 0.88 | -0.28 | 1.78 | 1.39E-05 |

^1^ Effect in standard deviation units of the transformed trait, thus a value of -0.22 indicates a decrease of 0.22 standard deviations with the presence of the + allele

^2^ Heritability reflects the proportion of the trait variance explained by the SNP

^┼^ Replication SNPs in GOLDN study

**Table S1**. **cont’d**

| **Trait** | **CHR** | **Position (bp)** | **SNP** | **Alleles  (+/-)** | **Freq (+)** | **EFFECT**^1^ | **H2**^2^ | **P-value** |
| --- | --- | --- | --- | --- | --- | --- | --- | --- |
| Alpha Linoleic Acid | 8 | 4803438 | rs10503296 | A/g | 0.70 | 0.22 | 2.07 | 2.71E-06 |
|  | 11 | 87027055 | rs11235247 | G/a | 0.98 | 0.65 | 1.98 | 3.80E-06 |
|  | 11 | 97400838 | rs593860 | G/a | 0.66 | 0.21 | 1.95 | 3.92E-06 |
|  | 6 | 136902424 | rs13191834 | T/c | 0.87 | -0.29 | 1.92 | 5.66E-06 |
|  | 17 | 54626861 | rs2291193 | G/a | 0.74 | -0.22 | 1.90 | 7.44E-06 |
|  | 8 | 26761498 | rs558455 | G/a | 0.50 | -0.19 | 1.84 | 1.12E-05 |
|  | 12 | 128660870 | rs265603 | A/c | 0.55 | -0.20 | 1.95 | 1.18E-05 |
|  | 18 | 3317801 | rs949306 | G/a | 0.77 | -0.21 | 1.71 | 1.31E-05 |
|  | 11 | 61362189 | rs174579 | C/t | 0.80 | -0.22 | 1.63 | 2.02E-05 |
|  | 6 | 35109114 | rs3800433 | C/t | 0.88 | 0.28 | 1.70 | 2.11E-05 |
| Eicosapentanoic Acid | 11 | 61326406 | rs174546 | C/t | 0.73 | 0.37 | 5.64 | 5.49E-15 |
|  | 4 | 188560638 | rs6553050 | T/c | 0.88 | -0.33 | 2.32 | 1.06E-06 |
|  | 6 | 11120845 | rs953413^┼^ | G/a | 0.57 | -0.21 | 2.24 | 1.10E-06 |
|  | 13 | 111744715 | rs9549825 | C/t | 0.69 | 0.21 | 1.97 | 4.24E-06 |
|  | 18 | 67854267 | rs2129588 | C/t | 0.65 | -0.20 | 1.83 | 8.93E-06 |
|  | 11 | 17041768 | rs7949405 | A/c | 0.80 | 0.24 | 1.93 | 9.48E-06 |
|  | 10 | 61333643 | rs1171826 | T/c | 0.64 | 0.20 | 1.83 | 1.30E-05 |
|  | 1 | 176896748 | rs3767165 | C/t | 0.65 | 0.20 | 1.94 | 1.37E-05 |
|  | 11 | 116090743 | rs2000571 | C/t | 0.77 | 0.23 | 1.85 | 1.42E-05 |
|  | 3 | 173791742 | rs11914753 | C/t | 0.97 | -0.53 | 1.77 | 3.13E-05 |
| Eicosapentanoic Acid | 12 | 56299442 | rs2277324^┼^ | A/g | 0.50 | 0.24 | 2.95 | 2.65E-08 |
|  | 17 | 22595293 | rs17703271 | T/c | 0.81 | 0.27 | 2.43 | 4.61E-07 |
|  | 18 | 40571788 | rs11082403 | T/g | 0.75 | 0.23 | 2.00 | 2.00E-06 |
|  | 1 | 202348506 | rs2153903 | G/a | 0.59 | 0.19 | 1.86 | 8.04E-06 |
|  | 7 | 85635018 | rs9969120 | A/g | 0.60 | 0.20 | 1.90 | 9.20E-06 |
|  | 6 | 39249191 | rs6921231 | A/g | 0.95 | 0.41 | 1.77 | 9.88E-06 |
|  | 8 | 134648275 | rs2272674 | A/g | 0.57 | 0.18 | 1.69 | 1.14E-05 |
|  | 20 | 15513140 | rs12481689 | A/g | 0.95 | 0.43 | 1.74 | 1.20E-05 |
|  | 4 | 20173546 | rs522501 | T/c | 0.75 | 0.21 | 1.67 | 1.56E-05 |
|  | 5 | 139146992 | rs749125 | C/t | 0.59 | 0.18 | 1.65 | 2.14E-05 |

^1^ Effect in standard deviation units of the transformed trait, thus a value of -0.22 indicates a decrease of 0.22 standard deviations with the presence of the + allele

^2^ Heritability reflects the proportion of the trait variance explained by the SNP

^┼^ Replication SNPs in GOLDN study
